# Rapid Mix Preparation of Bioinspired Nanoscale Hydroxyapatite for Biomedical Applications

**DOI:** 10.3791/55343

**Published:** 2017-02-23

**Authors:** Caroline J. Wilcock, Piergiorgio Gentile, Paul V. Hatton, Cheryl A. Miller

**Affiliations:** ^1^Bioengineering and Healthcare Technologies, School of Clinical Dentistry, University of Sheffield

**Keywords:** Bioengineering, Issue 120, Nanoscale, Hydroxyapatite, Calcium phosphate, Orthopaedic, Dental, Craniofacial, Bioinspired, Biomimetic

## Abstract

Hydroxyapatite (HA) has been widely used as a medical ceramic due to its good biocompatibility and osteoconductivity. Recently there has been interest regarding the use of bioinspired nanoscale hydroxyapatite (nHA). However, biological apatite is known to be calcium-deficient and carbonate-substituted with a nanoscale platelet-like morphology. Bioinspired nHA has the potential to stimulate optimal bone tissue regeneration due to its similarity to bone and tooth enamel mineral. Many of the methods currently used to fabricate nHA both in the laboratory and commercially, involve lengthy processes and complex equipment. Therefore, the aim of this study was to develop a rapid and reliable method to prepare high quality bioinspired nHA. The rapid mixing method developed was based upon an acid-base reaction involving calcium hydroxide and phosphoric acid. Briefly, a phosphoric acid solution was poured into a calcium hydroxide solution followed by stirring, washing and drying stages. Part of the batch was sintered at 1,000 °C for 2 h in order to investigate the products' high temperature stability. X-ray diffraction analysis showed the successful formation of HA, which showed thermal decomposition to β-tricalcium phosphate after high temperature processing, which is typical for calcium-deficient HA. Fourier transform infrared spectroscopy showed the presence of carbonate groups in the precipitated product. The nHA particles had a low aspect ratio with approximate dimensions of 50 x 30 nm, close to the dimensions of biological apatite. The material was also calcium deficient with a Ca:P molar ratio of 1.63, which like biological apatite is lower than the stoichiometric HA ratio of 1.67. This new method is therefore a reliable and far more convenient process for the manufacture of bioinspired nHA, overcoming the need for lengthy titrations and complex equipment. The resulting bioinspired HA product is suitable for use in a wide variety of medical and consumer health applications.

**Figure:**
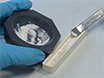


## Introduction

There is a great clinical need for advanced biomaterials with enhanced functionality in order to improve quality of life of patients and to reduce the healthcare burden of a global aging population. Hydroxyapatite has been widely used in medical applications for many years due to its good biocompatibility. Recently, there has been an increased interest in the use of nanoscale hydroxyapatite (nHA), particularly for mineralized tissue regeneration in medicine and dentistry. The mineral found in bone and tooth enamel is calcium-deficient, multi-substituted, nanoscale hydroxyapatite. Estimates for the size of biological nHA platelets report dimensions of 50 nm x 30 nm x 2 nm^1^, with even smaller structures described in immature bone^2^. Contrastingly, the mineral in tooth enamel is 10 to 100 times larger than that found in bone tissue in both length and width^3^,^4^. Synthetic nHA might be better termed bioinspired rather than biomimetic, as we are seeking to translate observations regarding the characteristics of natural materials into medical technologies with improved performance. It has been suggested that bioinspired nHA may be more favorable in bone and tooth tissue regeneration applications due to its similarity to naturally occurring mineral^5^.

There are various methods which have been reported to prepare nHA including hydrothermal^6^, spray-dry^7^ and sol-gel^8^ techniques. Of these, the wet precipitation method is considered a relatively convenient method for the production of nHA. The published nHA wet precipitation methods generally include a titration step when mixing calcium and phosphorus chemical precursors^9^,^10^,^11^,^12^,^13^,^14^. However, these approaches are associated with a number of disadvantages including lengthy and complex processes combined in some cases with the need for expensive equipment. Commercial production may be even more complex, with patents describing sophisticated reactors for manufacture of high quality medical grade nHA^15^. Despite this, the neutralization reaction between calcium hydroxide and phosphoric acid is advantageous due to the lack of noxious chemical by-products.

The relationship between processing conditions and the morphology of the nHA product has been reported for slow titration reactions. Specifically, for titration methods involving calcium hydroxide and phosphoric acid, an elevated temperature appeared to favor the preparation of particles with a low aspect ratio^13^. This work was extended considerably by Gentile *et al.*^16^ who demonstrated the relationship between temperature and other processing conditions on the quality of nHA products from a wide range of methods. He concluded that the wet chemical precipitation method of Prakash^13^ made the highest quality products, but it should be noted that the results were dependent upon technically challenging and slow/ mixing processes. The original Prakash titration step takes over one hour. However, longer titration times may be required for larger batches to be prepared.

To summarize, while the influence of several factors including temperature have now been studied extensively, almost no attention has been directed at reducing the complexity and associated time needed to perform titration-based methods. The aim of this study was therefore to investigate the effects of applying a rapid mix approach to the manufacture of a bioinspired nHA, and to fully characterize the resulting materials. If successful, a simplified rapid mix approach would have great benefits for laboratory researchers and industry alike where costs of manufacture could be substantially reduced without comprising quality.

## Protocol


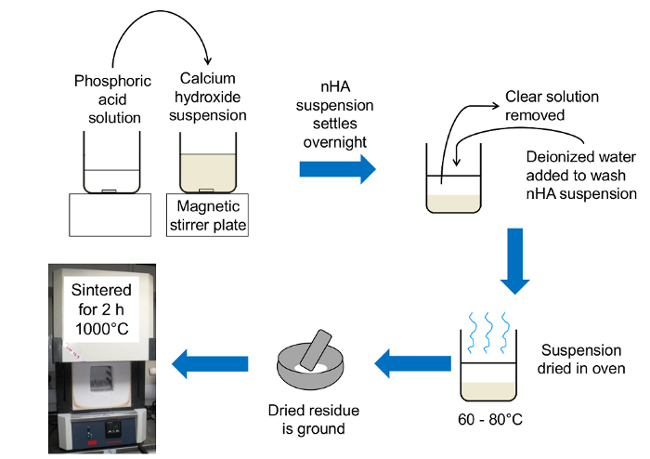
**Figure 1. Schematic diagram of rapid mix preparation of bioinspired nanoscale hydroxyapatite. **The phosphoric acid solution was poured into the calcium hydroxide suspension. After the suspension settled overnight, the nHA was washed with deionized water before being dried at 60 to 80°C. The nHA was then ground in an agate mortar and pestle and sintered to investigate the thermal stability of the nHA product. Please click here to view a larger version of this figure.

### 1. Rapid Mix Production of Nanoscale Hydroxyapatite

1. Preparation of calcium and phosphorus solutions to prepare 5 g of nanoscale hydroxyapatite using a calcium to phosphorus molar ratio of 1.67.
  1. Add 3.705 g of calcium hydroxide to 500 mL deionized water and stir on a magnetic stirrer plate for 1 h at 400 rpm.
  2. In a separate beaker, dissolve 3.459 g of phosphoric acid (85%) in 250 mL deionized water.
2. Pour the phosphorus solution into the stirring calcium hydroxide suspension at a rate of approximately 100 mL/s. Cover beaker with Parafilm (Bemis, USA).
3. Leave the suspension to stir for 1 h at 400 rpm.
4. Take the beaker off the stirrer plate and leave to settle overnight.
5. Wash the suspension by pouring off the supernatant and adding 500 mL deionized water and stirring for 1 min at 400 rpm. Repeat this step three times in total, with 2 h between each wash.
6. Leave nHA suspension to settle overnight.
7. Pour off the clear supernatant and place the settled nHA suspension in a drying oven set at 60 to 80 °C.
8. When dry, place the dried nHA into an agate mortar and pestle and grind until fine.
9. Place 2.5 g of produced nHA powder in an alumina crucible and sinter powder at 1,000 °C for 2 h using a ramp rate of 10 °C/min. After the heat treatment, leave the nHA to cool in the furnace.
10. Store powders in a vacuum desiccator.

### 2. Characterization of Nanoscale Hydroxyapatite

1. **X-ray diffraction (XRD) using transmission mode diffractometers**
  1. Place a small amount (*i.e.* less than 200 µL) of poly(vinyl alcohol) (PVA) glue on acetate film and mix with a small amount (*i.e.* less than 100 mg) of nHA powder.
  2. Treat with a hot air gun until dry.
  3. Mount the sample into a sample holder and load onto a transmission mode X-ray diffractometer with Cu K_α_ radiation.
  4. Use diffractometer settings of 40 kV and 35 mA, with a 2θ range of 10-70°.
  5. Analyze the resultant XRD patterns.
  6. Use the following XRD cards for phase identification: Hydroxyapatite: 9-432. β-tricalcium phosphate: 04-014-2292.
2. **Transmission electron microscopy (TEM)**
  1. Place a small amount of powder (*i.e.* less than 10 mg) in a bijou and add approximately 3 mL ethanol.
  2. Ultra-sonicate sample for 15 - 30 minutes until a homogenous suspension is observed.
  3. Pipette a small amount of solution (*i.e.* less than 1 mL) onto a 400 mesh copper grid with carbon film, and allow to dry.
  4. Image samples at an accelerating voltage of 80 kV.
3. **X-ray fluorescence (XRF) service by the Materials and Engineering Research Institute (MERI) at Sheffield Hallam University**
  1. Combine 0.8 g nHA powder with 8 g of lithium tetraborate.
  2. Melt mixture in a platinum-gold alloy crucible using a furnace set to 1,200 °C.
  3. Analyze resultant samples in an XRF spectrometer to determine the elemental composition of the samples.
4. **Fourier-transform infrared spectroscopy in attenuated total reflectance mode (FTIR-ATR)**
  1. Perform 64 background scans from 4,000 - 500 cm^-1^ with a resolution of 4 cm^-1^.
  2. Place a small amount (*i.e.* less than 100 mg) of nHA powder on top of the diamond in the attenuated total reflectance mode adapter and compress onto the surface of the diamond using the screw top.
  3. Perform 32 scans from 4,000 - 500 cm^-1^ with a resolution of 4 cm^-1 ^with the background scans subtracted from the sample scans.

## Representative Results

XRD patterns (**Figure 2**) showed the precipitation of a pure HA phase with broad peaks, indicating a relatively small crystallite size and/or amorphous nature. After high temperature sintering, β-tricalcium phosphate (β-TCP) was detected, alongside a main phase of HA. The sharpening of the diffraction peaks, *i.e.* a reduction in the full width half maximum, indicated an increase in the crystallite size after sintering.


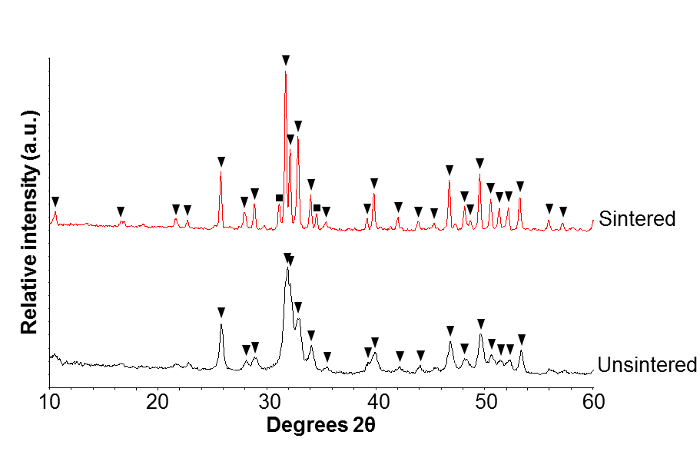
**Figure 2. Crystal phase analysis of products. **X-ray diffraction (XRD) patterns of unsintered nanoscale hydroxyapatite (nHA) powder and nHA powder sintered at 1,000 °C for 2 h. Peak labels: ▼ hydroxyapatite peaks, ■ β-tricalcium phosphate peaks. Please click here to view a larger version of this figure.

FTIR-ATR spectra (**Figure 3**) confirmed the formation of a HA phase by the characteristic phosphate and hydroxyl bands^17^,^18^. In detail the bands were assigned as follows: 3,750 cm^-1^ (OH^-^ stretch ν_OH_); 1,086 and 1,022 cm^-1^ (PO_4_^3-^ ν_3_); 962 cm^-1^ (PO_4_^3-^ ν_1_); 630 cm^-1^ (OH^-^ libration δ_OH_); 600 and 570 cm^-1^ (PO_4_^3-^ ν_4_). In the unsintered sample the additional peaks were assigned as follows: broad peak centered around 3,400 cm^-1^ (absorbed water molecules); 1,455 and 1,410 cm^-1^ (CO_3_^2-^ ν_3_); 880 cm^-1^ (CO_3_^2-^ ν_2_). The absorbed water and carbonate groups observed in the unsintered powder were removed during the high temperature sintering stage. The sintering process also sharpened the hydroxyl and phosphate bands which was manifested by a greater peak to trough distance.


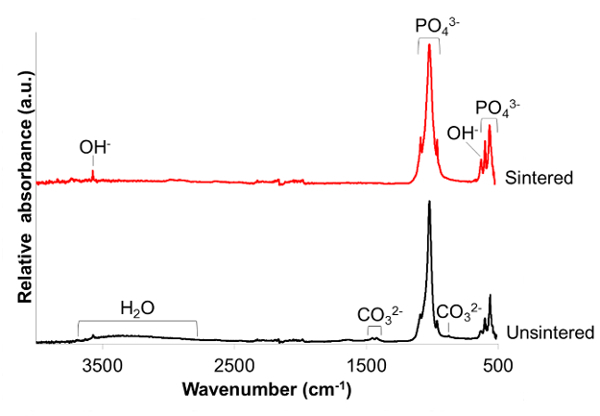
**Figure 3. Infrared spectra of products. **Fourier transform infrared in attenuated total reflectance mode (FTIR-ATR) spectra of unsintered nanoscale hydroxyapatite (nHA) powder and nHA powder sintered at 1,000 °C for 2 h. Please click here to view a larger version of this figure.

TEM images (**Figure 4**) showed the formation of nanoscale particles with approximate dimensions of 50 nm by 30 nm. The particles had a low aspect ratio (particle length / particle width) of around 1.7. The size and shape of the nanoscale products were of similar dimensions to biological apatite^1^.


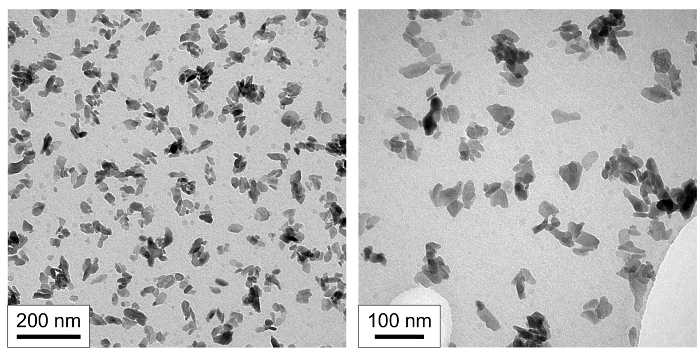
**Figure 4. Nanoscale morphology of product. **Transmission electron micrographs (TEM) of nanoscale hydroxyapatite (nHA) prepared using the rapid mixing method at two magnifications. Please click here to view a larger version of this figure.

Quantitative chemical analysis of the nHA powder by XRF (**Table 1**) allowed the calcium: phosphorus ratio to be calculated as 1.63, which is slightly lower than the stoichiometric HA which has a calcium: phosphorus ratio of 1.67. XRF also showed the high purity of the nHA product with only trace amounts of other elements recorded.

**Table:** 

**Compound**	**Weight %**
CaO	51.52
P_2_O_5_	39.89
MgO	0.46
Na_2_O	0.13
Y_2_O_3_	0.07
Al_2_O_3_	0.03
SiO_2_	0.03
Mn_3_O_4_	0.03
SrO	0.02
TiO_2_	0.01

**Table 1. Quantitative chemical analysis of product. **X-ray fluorescence (XRF) results for unsintered nHA powder showed >99% purity by weight.

## Discussion

Natural apatite is composed of nanoscale particles of non-stoichiometric carbonated hydroxyapatite with the approximate chemical formula of Ca_10-x-y_[(HPO_4_)(PO_4_)]_6-x_(CO_3_)_y_(OH)_2-x_. The production of biomaterials with close chemical similarity to naturally occurring mineral has been reported to promote optimal biological responses. For instance, research on biomimetic calcium-deficient carbonated nHA has shown it is able to stimulate proliferation and the alkaline phosphatase activity of murine preosteoblast cells to a greater degree than conventional nHA^19^.

In this study, the precipitation of HA which showed partial thermal decomposition at 1,000 °C (**Figure 2**) suggested the formation of a calcium-deficient HA. This was supported by the lower than stoichiometric Ca:P ratio (1.63) obtained with the XRF data (**Table 1**). It is understood that a reduced Ca:P ratio is associated with a lower thermal stability^20^,^21^,^22^,^23^. In this method, the rapid addition of the phosphoric acid solution rapidly lowered the pH of the reaction suspension to generate HPO_4_ ions. The presence of HPO_4_ groups facilitated the precipitation of calcium deficient HA, with the molecular formula: Ca_10-x_(HPO_4_)_ x_(PO_4_)_6-x_(OH)_2-x_, where 0<x<1.

The rapid addition of the phosphoric acid therefore had a marked effect on the precipitation kinetics of the reaction. As described previously, titration reactions involving calcium hydroxide and phosphoric acid carried out at room temperature tended to yield particles with a high aspect ratio^13^. For titration reactions involving these reactants, it was necessary to use an elevated temperature to produce particles with a lower aspect ratio which are more similar to biological apatite^13^. High aspect ratio particles are produced when the crystal nucleation rate is slower than the crystal growth rate^24^. For the new method developed in this study, the rapid addition of the phosphoric acid solution may have provided a larger number of nucleation sites which resulted in the increased presence of small rounded particles as opposed to fewer particles with a larger aspect ratio. As the authors have not fully investigated the effects of slowly pouring the phosphoric acid into the calcium hydroxide suspension, in order to achieve consistent results we recommend that the phosphoric acid is poured at a rate commensurate with that shown in the video (approximately 100 mL/s).

During the development of this method, the authors investigated a number of incremental changes to the nHA preparation method based on Prakash *et al*.^13^ including the comparison of products produced with the slow titration and the rapid addition of the phosphoric acid solution^25^. It was found that the slow titration of phosphoric acid into the calcium hydroxide suspension resulted in a product with a calcium hydroxide residue. We propose that the pH change caused by the rapid addition of phosphoric acid encouraged the dissolution of the calcium hydroxide and therefore allowed for the successful conversion of the reactants into hydroxyapatite. A comparison of products prepared using the rapid mixing method at room and elevated temperatures (60 °C) found that an elevated temperature resulted in a higher conductivity after the reaction was completed. This suggested that residual calcium hydroxide was present which was likely to be due to the lower solubility of calcium hydroxide at increased temperatures. The presence of residual calcium hydroxide was undesirable as the basic nature of this compound could compromise biocompatibility.

FTIR detected the characteristic phosphate and hydroxyl group activity associated with HA (**Figure 3**). It was noted that the spectrum for the sintered product showed sharper phosphate and hydroxyl peaks. These changes have been associated with a greater product crystallinity^26^,^27^.The unsintered spectrum provided evidence for B-type carbonate substitution where carbonate ions have substituted for phosphate groups. This is in contrast to A-type substitution where carbonate ions may substitute for hydroxyl groups^17^. It has been reported that B-type carbonate substitution occurs in biological apatite^3^. However, Tampieri *et al. *reported that whilst B-type substitution was predominant in young bones, A-type carbonate substitution was increasingly present in bones of older individuals^28^. Carbonate substitution has been found to decrease the crystallinity and thermal stability of the nHA whilst increasing its solubility. These changes have been proposed to contribute to the increased bioactivity of carbonate-substituted HA^29^. Biological HA is also known to contain some of the other elements recorded in the XRF analysis (**Table 1**), such as magnesium, sodium and strontium^30^. The presence of these elements may also contribute to increased biological efficacy. Future work should be directed at the preparation of these nanoscale substituted apatites, and also products with increased biofunctionality such as silver-doped nHA^31^. In order to prepare substituted nHA, the element may be introduced with a corresponding reduction of the intended element to substitute for, *e.g.* a reduction in the amount of the calcium compound when strontium, magnesium or zinc substitution is attempted^32^. Alternatively, another approach may be to add elements with the intention of providing 'doped' ions which are present on the surface of the nHA without necessarily intending to substitute the element into the HA crystal lattice^31^. For these modifications to the method it is possible to prepare mixed solutions such as calcium hydroxide and silver nitrate, and to carry out the reaction in the same manner as described here.

In conclusion, this paper reports a novel rapid and substantially improved method for the preparation of bioinspired nHA. For this method, the rapid mixing of the chemicals takes less than 5 seconds which is a marked reduction in time compared to titrations reactions typically requiring hours of careful monitoring. It has great potential for use in biomaterial development due to its relative simplicity and low cost compared to currently used industrial nHA manufacturing methods where the inherent complexity of current commercial systems results in lengthy research and development times, and substantially increased manufacturing costs. In particular, this new method is superior to continuous flow processes or hydrothermal techniques due to significantly lower start-up equipment investment requirements.

## Disclosures

The authors have nothing to disclose.

## References

[B0] Pasteris JD, Wopenka B, Valsami-Jones E (2008). Bone and tooth mineralization: why apatite?. Elements.

[B1] Carter DH, Hatton PV, Aaron JE (1997). The ultrastructure of slam-frozen bone mineral. Histochem. J.

[B2] Wopenka B, Pasteris JD (2005). A mineralogical perspective on the apatite in bone. Mater. Sci. Eng.

[B3] Boskey AL (2007). Mineralization of bones and teeth. Elements.

[B4] Fox K, Tran PA, Nhiem T (2012). Recent Advances in Research Applications of Nanophase Hydroxyapatite. ChemPhysChem.

[B5] Neira IS (2009). An Effective Morphology Control of Hydroxyapatite Crystals via Hydrothermal Synthesis. Cryst. Growth. Des.

[B6] Luo P, Nieh TG (1995). Synthesis of ultrafine hydroxyapatite particles by a spray dry method. Mater. Sci. Eng. C.

[B7] Wang F, Li MS, Lu YP, Qi YX (2005). A simple sol-gel technique for preparing hydroxyapatite nanopowders. Mater. Lett.

[B8] Cai Y (2007). Role of hydroxyapatite nanoparticle size in bone cell proliferation. J. Mater. Chem.

[B9] Catros S (2010). Physico-chemical and biological properties of a nano-hydroxyapatite powder synthesized at room temperature. IRBM.

[B10] Kumar R, Prakash KH, Cheang P, Khor KA (2004). Temperature driven morphological changes of chemically precipitated hydroxyapatite nanoparticles. Langmuir.

[B11] Liu H, Yazici H, Ergun C, Webster TJ, Bermek H (2008). An in vitro evaluation of the Ca/P ratio for the cytocompatibility of nano-to-micron particulate calcium phosphates for bone regeneration. Acta. Biomater.

[B12] Prakash KH, Kumar R, Ooi CP, Cheang P, Khor KA (2006). Apparent solubility of hydroxyapatite in aqueous medium and its influence on the morphology of nanocrystallites with precipitation temperature. Langmuir.

[B13] Bianco A, Cacciotti I, Lombardi M, Montanaro L, Gusmano G (2007). Thermal stability and sintering behaviour of hydroxyapatite nanopowders. J. Therm. Anal. Calorim.

[B14] Brito Lopes JC (2008). Production method for calcium phosphate nano-particles with high purity and their use. WO2008/007992A2.

[B15] Gentile P, Wilcock CJ, Miller CA, Moorehead R, Hatton PV (2015). Process optimisation to control the physico-chemical characteristics of biomimetic nanoscale hydroxyapatites prepared using wet chemical precipitation. Materials.

[B16] Gibson IR, Bonfield W (2002). Novel synthesis and characterization of an AB-type carbonate-substituted hydroxyapatite. J. Biomed. Mater. Res.

[B17] Koutsopoulos S (2002). Synthesis and characterization of hydroxyapatite crystals: a review study on the analytical methods. J. Biomed. Mater. Res.

[B18] Deng Y, Sun Y, Chen X, Zhu P, Wei S (2013). Biomimetic synthesis and biocompatibility evaluation of carbonated apatites template-mediated by heparin. Mater. Sci. Eng. C.-Mater. Biol. Appl.

[B19] Gibson IR, Rehman I, Best SM, Bonfield W (2000). Characterization of the transformation from calcium-deficient apatite to beta-tricalcium phosphate. J. Mater. Sci.-Mater. M.

[B20] Siddharthan A, Seshadri SK, Kumar TSS (2004). Microwave accelerated synthesis of nanosized calcium deficient hydroxyapatite. J. Mater. Sci.-Mater. M.

[B21] Yubao L, Klein C, Dewijn J, Vandemeer S, Degroot K (1994). Shape change and phase-transition of needle-like nonstoichiometric apatite crystals. J. Mater. Sci.-Mater. M.

[B22] Prieto Valdes JJ, Ortiz Lopez J, Rueda Morales G, Pacheco Malagon G, Prieto Gortcheva V (1997). Fibrous growth of tricalcium phosphate ceramics. J. Mater. Sci.-Mater. M.

[B23] Bouyer E, Gitzhofer F, Boulos MI (2000). Morphological study of hydroxyapatite nanocrystal suspension. J. Mater. Sci.-Mater. M.

[B24] Wilcock CJ (2015). The development of nanostructured calcium phosphate biomaterials for bone tissue regeneration PhD thesis.

[B25] Khalid M (2013). Effect of surfactant and heat treatment on morphology, surface area and crystallinity in hydroxyapatite nanocrystals. Ceram. Int.

[B26] Reyes-Gasga J (2013). XRD and FTIR crystallinity indices in sound human tooth enamel and synthetic hydroxyapatite. Mater. Sci. Eng. C.-Mater. Biol. Appl.

[B27] Tampieri A, Celotti G, Landi E (2005). From biomimetic apatites to biologically inspired composites. Anal. Bioanal. Chem.

[B28] Boanini E, Gazzano M, Bigi A (2010). Ionic substitutions in calcium phosphates synthesized at low temperature. Acta. Biomater.

[B29] Elliott JC (1994). Structure and Chemistry of the Apatites and Other Calcium Orthophosphates.

[B30] Wilcock CJ (2017). Preparation and Antibacterial Properties of Silver-doped Nanoscale Hydroxyapatite Pastes for Bone Repair and Augmentation. J. Biomed. Nanotechnol.

[B31] Cox SC, Jamshidi P, Grover LM, Mallick KK (2014). Preparation and characterisation of nanophase Sr, Mg, and Zn substituted hydroxyapatite by aqueous precipitation. Mater. Sci. Eng. C.

